# Mean platelet volume/platelet count ratio can predict the recurrence-free survival rate of patients after complete resection of gastrointestinal stromal tumors

**DOI:** 10.3389/fonc.2024.1465283

**Published:** 2024-11-08

**Authors:** Xinlian Du, Xinxin Zang, Hanbo Zhang, Lijia Liu, Ying Xu, Xuedong Li, Ruishu Mou, Haitao Xu, Jiuxin Zhu, Rui Xie

**Affiliations:** ^1^ Department of Digestive Internal Medicine, Harbin Medical University Cancer Hospital, Harbin Medical University, Harbin, Heilongjiang, China; ^2^ Department of Hepatobiliary and Pancreatic Surgery, Harbin Medical University Cancer Hospital, Harbin Medical University, Harbin, Heilongjiang, China; ^3^ College of Pharmacy, Harbin Medical University, Harbin, Heilongjiang, China

**Keywords:** mean platelet volume/platelet count ratio, PVPR, recurrence-free survival, gastrointestinal stromal tumors, prognosis, nomogram

## Abstract

**Purpose:**

The aim of this study is to compare mean platelet volume/platelet count ratio (PVPR) and other indicators’ predictive abilities. Simultaneously, a new nomogram for predicting recurrence-free survival (RFS) after gastrointestinal stromal tumors (GISTs) R0 resection was developed.

**Methods:**

From January 2010 to July 2019, 295 patients with GIST who were operated on at Harbin Medical University Cancer Hospital were retrospectively reviewed. With a 4-year RFS as the end point, using the Kaplan–Meier methods and log rank test, and then conducting Cox regression analysis, we compared and identified meaningful indicators for predicting prognosis. Finally, a nomogram was developed and validated using calibration curves.

**Results:**

The receiver operating characteristic curve indicated that a cutoff point of 0.044 was the ideal threshold for PVPR, and patients were divided into a high-PVPR group (≤0.044) and a low-PVPR group (>0.044). Kaplan–Meier curves suggested that PVPR>0.044 had obvious associations with better RFS (p < 0.001). In accordance with multivariate analysis, PVPR (>0.044 vs. ≤0.044) (p = 0.005), National Institutes of Health (NIH) risk category (p < 0.001), and Ki-67 (p = 0.005) were the independent prognostic indicators of RFS. Tumor size, gastrointestinal bleeding, mitotic index, NIH risk category, CD34, and Ki-67 all exhibited an obvious correlation with PVPR (all p < 0.05). The nomogram’s probability of concordance was 0.823, indicating that the nomogram predictions were well calibrated.

**Conclusion:**

In GISTs, RFS can be independently predicted by PVPR. Patients with higher PVPR have better RFS. The nomogram including PVPR could be used to assist clinical treatment decision-making.

## 1 Introduction

Gastrointestinal stromal tumors (GISTs) are one of the most frequently occurring sarcomas. Typically, GISTs originate from the stromal cells of Cajal, in which the occurrence rate is relatively low in the population (1) and the most common cause is mutations in receptor tyrosine kinases, especially in people with KIT proto-oncogene receptor tyrosine kinase (KIT) or Platelet-derived growth factor receptor alpha (PDGFRA) gene mutations (2). GISTs have been reported case in all age groups and are common in adults over 40 years old (3). It can appear everywhere in the digestive tract and usually in the stomach (60%–65%); next is the small intestine (20%–25%); a few are found in areas outside the gastrointestinal tract, such as the peritoneum, mesentery, and omentum. The clinical manifestations of GIST are non-specific, and bleeding, pain, and obstruction are common clinical manifestations (4). Comprehensive judgment is required on the basis of imaging examination, endoscopic examination, pathological tissue, immunohistochemistry, and genetic protein. The classification of GIST mainly applies NIH risk classification (5). GIST treatment includes endoscopic treatment, surgical treatment, and medication treatment. Small molecular tyrosine kinase inhibitors are the most frequently used therapeutic drugs of GISTs, and patients’ long-term prognosis can be vastly enhanced (6). However, patients with GIST still have a certain recurrence rate after surgery.

Currently, some GIST basic indicators, such as primary tumor location, mitotic index, tumor size, and tumor rupture, have been applied to estimate the probabilities of GIST recurrence (7, 8). Although the results obtained from the risk classification of NIH are identical, the prognosis of GIST varies significantly. Moreover, these indicators require invasive examination to obtain. Therefore, discovering a simple, non-invasive, acceptable, and affordable indicator to direct the treatment plan and pinpoint patients who are at a greater risk of recurrence is very necessary.

Nowadays, an essential part of the pathogenesis of malignant tumors is inflammation.

Chronic systemic inflammation is closely correlated to the long-term prognosis of many kinds of cancers. In addition to promoting tumor cells to elude immune monitoring, systemic inflammation can compromise the body’s immune response and accelerate angiogenesis, invasion, and metastasis (9). Some markers of systemic inflammation, such as Lymphocyte - to - White Blood Cell Ratio (LWR) and Systemic Inflammatory Response Index (SIRI), are based on lymphocyte, neutrophils, and monocyte, previous studies have confirmed their prognostic potential in malignant tumors (10, 11). Meanwhile, some studies have analyzed the ability of inflammatory markers such as platelet volume/platelet count ratio (PVPR), High - Reactivity Platelet - related factor (HPR), and Red Cell Platelet Ratio (RPR) based on hemoglobin, red cells, and platelets to reflect the impact of platelet activation on the progression of malignant tumors (12, 13). A number of studies to date have suggested that PVPR is associated with a wide range of tumors and that reduced PVPR is a hallmark of poor chemotherapy outcomes in advanced Gastric Cancer (GC) and is associated with long PFS in glioblastoma, as well as poor lung cancer diagnosis and reduced overall survival (14–16). However, it is unclear whether PVPR is associated with the risk of postoperative recurrence in patients with GIST. Therefore, we evaluated the prognostic value of PVPR, LWR, HPR, SIRI, and RPR in GISTs and contrasted their ability to forecast survival in this research.

## 2 Materials and methods

### 2.1 Patients

**Figure 1:**
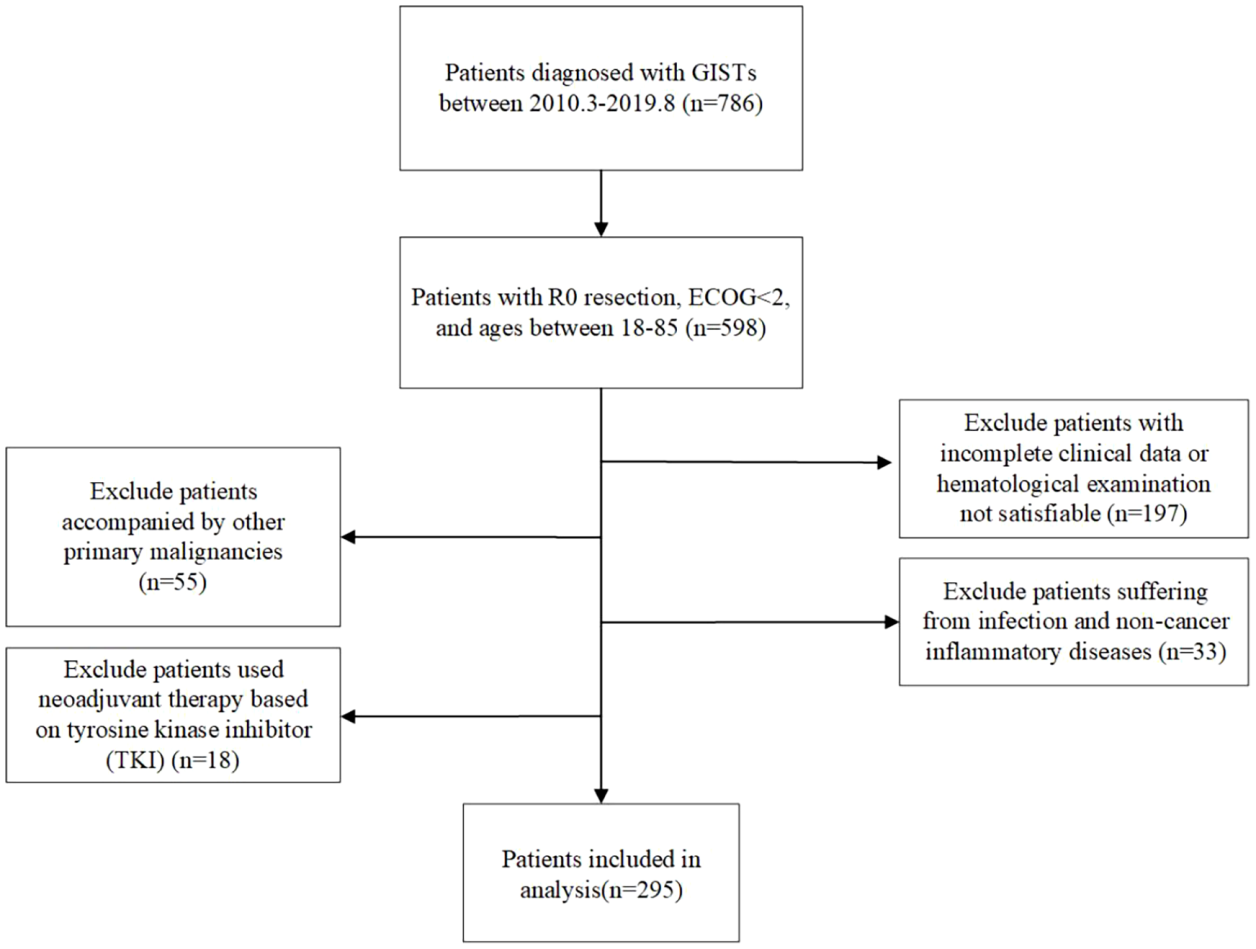
The flowchart of this study.

This study retrospectively analyzed patients with GIST who underwent surgical treatment at the Cancer Hospital of Harbin Medical University from January 2010 to July 2019. Each individual was pathologically given a GIST diagnosis and underwent R0 resection surgery with ECOG <2, aged between 18 and 85 years old. Patients who had incomplete preoperative clinical records or hematological examination data, simultaneously suffering from other tumors, experiencing infection and non-cancerous inflammatory disorders, and utilizing tyrosine kinase inhibitor (TKI)–based neoadjuvant treatment, were excluded. In total, 295 patients with GIST were ultimately included in this retrospective study (**Figure 1**). This retrospective study was approved by the Ethics Committee of Harbin Medical University Cancer Hospital, and all patients signed informed consent forms. This study complies with the Helsinki Declaration.

### 2.2 Indicators

The fundamental patient parameters, such as age, gender, tumor site, NIH risk category, tumor size, and mitotic index [mitosis/50 high-power field (HPF)], morphology, immunohistochemistry, molecular markers were recorded. The period elapsed between surgery and the first recorded occurrence of tumor or death is known as the recurrence-free survival (RFS). The data were collected from peripheral blood tests 2 weeks before surgery, including neutrophils, lymphocytes, white blood cells, platelet counts (PCs), hemoglobin, mean platelet volume (MPV), and red cell distribution width and monocyte count. The PVPR, LWR, HPR, SIRI, and RPR were calculated using the following formulas: PVPR = MPV (fL)/PC (10^9^/L); LWR = lymphocyte numbers (10^9^/L)/white blood cells (10^9^/L); HPR = hemoglobin (g/L)/PC (10^9^/L); SIRI = neutrophils (10^9^/L) × monocyte count (10^9^/L)/lymphocyte count (10^9^/L); RPR = red cell distribution width/PC (10^9^/L). In this trial, no patient passed away within 30 days following surgery.

### 2.3 Follow-up

During the first 3 years following the procedure, abdominal/pelvic computed tomography or magnetic resonance imaging was done every 3 to 6 months. After that, it was done every 6 to 12 months until 5 years later and then once a year until recurrence. The last follow-up date was July 2023.

### 2.4 Statistical analysis

We determined the ideal cutoff values of PVPR, LWR, HPR, SIRI, and RPR by using the maximum value of the Youden index (sensitivity + specificity − 1) in accordance with the receiver operating characteristic (ROC) curve for predicting 4-year RFS (14).

After that, we calculated the area under the curve (AUC) to compare the biomarkers’ projected values. We split the patients into two groups on the basis of the cutoff values of each indicator. Continuous variables that conform to a normal distribution are represented by the mean ± standard deviation, whereas, conversely, the median and quartile are used. The categorical variables are displayed as absolute values and analyzed using chi-square tests. We use Kaplan–Meier method to calculate the survival curve of RFS and compare it through log-rank tests. Using the Cox proportional hazards regression model and the stepwise forward method for variable selection, we performed univariate and multivariate analyses of survival. The hazard ratio (HR), which is determined as relative risk based on Cox analysis, was reported along with its associated 95% confidence interval (CI). Inflammatory markers with a P-value less than 0.05 were considered statistically significant in univariate analysis and designated for further multivariate analysis.

We constructed a nomogram using the independent risk variables of RFS discovered using multivariate Cox regression analysis. To evaluate nomogram’s performance, we employed a calibration curve on the basis of the Bootstrap sampling, which was repeated 1,000 times. The Harrell consistency index (C-index) is used to evaluate the predictive ability of column chart models. We used SPSS (version 25.0), GraphPad Prism 9.0, and R (version 4.3.1). The R packages rms, survival, mstate, Hmisc, dcurves, and ggplot2 (available at URL: http://cran.r-project.org/web/packages/) were used to do analysis of data.

## 3 Results

### 3.1 Optimal cutoff value of inflammatory indicators

**Figure 2:**
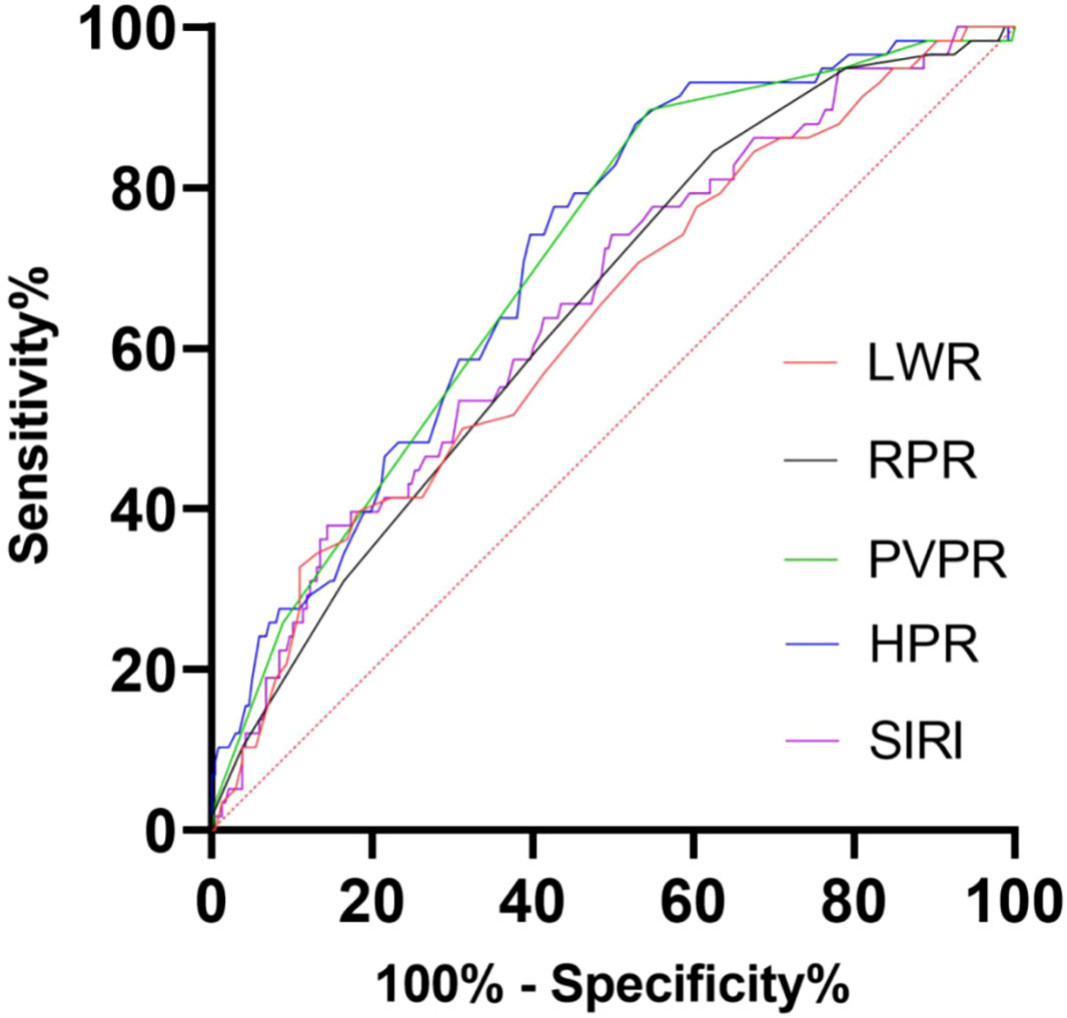
The receiver operating characteristic (ROC) analysis of LWR, RPR, PVPR, HPR, and SIRI. The areas under the curve (AUC) for RFS were 0.636 (p = 0.001), 0.662 (p < 0.001), 0.721 (p < 0.001), 0.713 (p < 0.001), and 0.655 (p < 0.001) for LWR, RPR, PVPR, HPR, and SIRI, respectively.

We determined the PVPR, LWR, HPR, SIRI, and RPR best cutoff values utilizing the ROC curves. Our results indicated that the optimal cutoff values of PVPR, LWR, HPR, SIRI, and RPR for predicting RFS were 0.044, 0.218, 0.607, 0.745, and 0.058, respectively. Patients were divided into two groups (PVPR >0.044 vs. ≤0.044; LWR >0.218 vs. ≤0.218; HPR >0.607 vs. ≤0.607; SIRI >0.745 vs. ≤0.745; RPR >0.058 vs. ≤0.058) for RFS analysis. Their AUC values are 0.721, 0.636, 0.713, 0.655, and 0.662, respectively, and all of their p-values are less than 0.001. PVPR outperformed LWR, HPR, RPR, or SIRI in terms of prognostic usefulness for patients with GIST, as evidenced by its higher AUC values when forecasting the 4-year survival rates for GISTs in comparison to other systemic inflammatory biomarkers (**Figure 2**).

### 3.2 Patients and basic characteristics

**Table 1** enumerates the clinical features. We accepted 295 individuals according to the inclusion criteria, including 135 men (45.8%) and 160 women (54.2%), and the ratio is approximately 1:1. Ages varied from 28 to 84, with stomach being the most often seen tumor location (198, 67.1%), then the small intestine (66, 22.4%), and colorectum (11, 3.7%); some are located extra-gastrointestinal 20 (6.8%), including esophagus, peritoneum, abdominal and pelvic cavity, pancreas, and liver. According to tumor size, the tumor’s maximal diameter on average was 6.0 cm. Patients were categorized into ≤4-cm, 4.1- to 6-cm, 6.1- to 9-cm, and ≥9-cm groups; each group has 93 (31.5%), 73 (24.7%), 72 (24.4%), 57 (19.4%) people, respectively. Most patients accepted open surgery (255, 86.4%). On the basis of the clinical data, 61 (20.7%) patients had gastrointestinal bleeding. According to the updated NIH GIST risk categorization standards, 108 (36.6%) patients were high risk, 73 (24.7%) patients were intermediate risk, 91 (30.8%) patients were low risk, and 23 (7.9%) patients were very low risk. For the majority of patients (214, 72.5%), histologic subtypes were spindle; the rest were mixed (72, 24.4%) and epithelioid (9, 3.1%). According to our research, whether the patients are using imatinib or sunitinib was investigated, up to 120 patients who explicitly used postoperative adjuvant were successfully followed up, and common adverse reactions include systemic or local edema gastrointestinal reaction and rash.

**Table 1:** Baseline characteristics of the patients with GIST enrolled in the study.

Characteristics	Total (n=295)
Age (median range, years) (%)	60 (28-84)
≤60	164 (55.6)
>60	131 (44.4)
Sex (%)	
Male	135 (45.8)
Female	160 (54.2)
Tumor site (%)	
Stomach	198 (67.1)
Colorectum	11 (3.7)
Small intestine	66 (22.4)
Other	20 (6.8)
Tumor size (cm) (%)	
≤4.0	93 (31.5)
4.1-6.0	73 (24.7)
6.1-9.0	72 (24.4)
>9.0	57 (19.4)
Surgery (%)	
Open	255 (86.4)
Minimally invasive	40 (13.6)
Gastrointestinal Bleeding (%)	
Yes	61 (20.7)
No	234 (79.3)
Mitotic index/50 HPF (%)	
≤5	225 (76.3)
6-10	54 (18.3)
>10	10 (3.4)
Unknown	6 (2.0)
NIH risk category (%)	
High risk	108 (36.6)
Intermediate risk	73 (24.7)
Low risk	91 (30.8)
Very low risk	23 (7.9)
Histologic subtypes (%)	
Spindle	214 (72.5)
Epithelioid	9 (3.1)
Mixed	72 (24.4)
Adjuvant therapy (%)	
Yes	120(40.7)
No	84 (28.5)
Unknown	91 (30.8)
CD117 (%)	
(+)	283 (95.9)
(+, partial)	10 (3.4)
(-)	2 (0.7)
CD34 (%)	
(+)	239 (81.0)
(+, partial)	24 (8.1)
(-)	32 (10.9)
DOG-1 (%)	
(+)	278 (94.2)
(+, partial)	7 (2.4)
(-)	10 (3.4)
SMA (%)	
(+)	38 (12.9)
(+, partial)	33 (11.2)
(-)	224 (75.9)
Desmin (%)	
(+)	10 (3.4)
(+, partial)	4 (1.3)
(-)	281 (95.3)
Ki-67 (%)	
≤10	218 (73.9)
>10	39 (13.2)
Unknown	38 (12.9)
Lymphocyte (mean ± SD)	1.77±0.59
Platelet (median [IQR])	243.00 (196.00,295.00)
Monocyte (mean ± SD)	0.40 (0.32,0.50)
Hemoglobin (mean ± SD)	124.22±26.69
Mean platelet volume (mean ± SD)	9.65±1.37
RPR (median [IQR])	0.06 (0.05,0.07)
PVPR (median [IQR])	0.04 (0.03,0.05)
HPR (median [IQR])	0.55 (0.37,0.69)
LWR (mean ± SD)	0.31±0.09
SIRI (median [IQR])	0.81 (0.49,1.20)
Recurrence (%)	
Yes	58 (80.3)
No	237 (19.7)

### 3.3 Univariate and multivariate survival analyses

**Figure 3:**
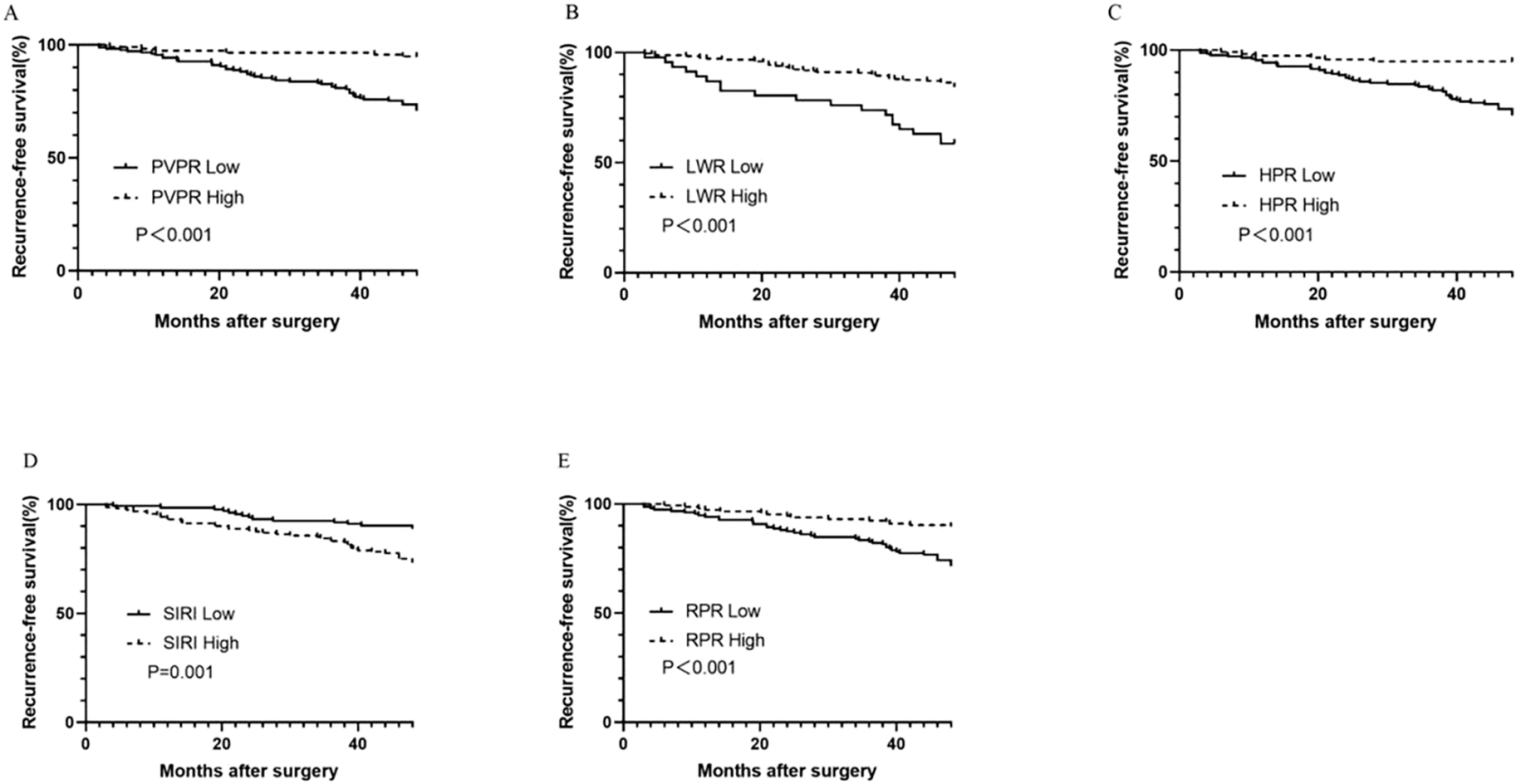
Kaplan–Meier survival curves for RFS according to PVPR **(A)**, LWR **(B)**, HPR **(C)**, SIRI **(D)**, and RPR **(E)** in patients with GIST.

The predictive value of inflammatory biomarkers was examined using univariate analysis and Kaplan–Meier survival analysis. The Kaplan–Meier survival curves of the PVPR, LWR, HPR, SIRI, and RPR indices are presented in **Figures 3A–E**. Compared to that in the PVPR-low group, the RFS rate in the PVPR-high group was much greater. Moreover, the results of the other four indicators in evaluating the prognosis of patients with GIST are statistically significant. **Table 2** presents the results of the univariate and multivariate analyses. The results of the univariate analyses showed that the mitotic index (P < 0.001), tumor size (P < 0.001), tumor site (P = 0.01), NIH risk category (P < 0.001), surgery (P = 0.028), adjuvant therapy (P = 0.003), CD34 (P = 0.003), DOG-1 (P = 0.001), Ki-67 (P < 0.001), RPR (HR = 0.334, 95% CI: 0.186–0.601, P < 0.001), PVPR (HR = 0.155, 95% CI: 0.066–0.360, P < 0.001), HPR (HR = 0.155, 95% CI: 0.066–0.360, P < 0.001), LWR (HR = 0.316, 95% CI: 0.183–0.548, P < 0.001), and SIRI (HR = 2.601, 95% CI: 1.445–4.682, P = 0.001) were remarkable predictors of RFS. The Cox multivariate analysis, which selected variables using a forward stepwise method, revealed that the NIH risk category (P < 0.001), Ki-67 (P = 0.005), and PVPR (HR = 0.281, 95% CI: 0.117–0.675, P = 0.005) were the independent prognostic factors of RFS.

**Table 2:** Univariate and multivariate analysis of the prognostic factors for recurrence-free survival in patients with GIST.

Variables	Univariate			Multivariate		
	HR	P-value	95%CI	HR	95%CI	P value
Age						
≤60vs>60	1.614	0.962-2.708	0.070			
Sex						
Male vs Female	0.909	0.540-1.528	0.718			
Tumor site			0.010			
Stomach	1.000					
Colorectum	0.557	0.076-4.088	0.565			
Small intestine	2.390	1.368-4.176	0.002			
Other	2.171	0.903-5.216	0.083			
Tumor size (cm)			<0.001			
≤4.0	1.000					
4.1-6.0	2.008	0.715-5.642	0.186			
6.1-9.0	4.490	1.793-11.245	0.001			
>9.0	8.671	3.541-21.231	<0.001			
Surgery						
Open vs Minimally invasive	4.886	1.192-20.021	0.028			
Gastrointestinal Bleeding	1.536	0.863-2.731	0.144			
Yes vs No			<0.001			0.068
Mitotic index/50 HPF	1.000			1.000		
≤5	3.839	2.142-6.883	<0.001	0.955	0.480-1.900	0.896
6-10	6.726 30.658	2.760-16.387 12.026-78.159	<0.001 <0.001	0.865 3.998	0.319-2.343 3.998-1.430	0.776 0.008
>10			<0.001			<0.001*
Unknown	1.000			1.000		
NIH risk category	0.239	0.117-0.489	<0.001	0.449	0.199-1.013	0.054
High risk	0.082	0.067-0.255	<0.001	0.146	0.048-0.444	0.001
Intermediate risk	0.000	0.000	0.971	0.000	0.000-9.752E+209	0.960
Low risk			0.051			
Very low risk	1.000					
Histologic subtypes	3.367	1.200-9.450	0.021			
Spindle	1.417	0.798-2.516	0.235			
Epithelioid						
Mixed						
Adjuvant therapy			0.003			
Yes	1.000					
No	2.624	1.283-5.368	0.008			
unknown	3.210	1.619-6.363	0.001			
CD117			0.460			
(+)	1.000					
(+, partial)	0.502	0.070-3.628	0.495			
(-)	2.830	0.391-20.463	0.303			
CD34			0.003			
(+)	1.000					
(+, partial)	0.699	0.217-2.255	0.549			
(-)	2.811	1.508-5.239	0.001			
DOG-1			0.001			
(+)	1.000					
(+, partial)	0.000	0.000-2.411E+220	0.965			
(-)	4.848	2.075-11.324	<0.001			
SMA			0.614			
(+)	1.000					
(+, partial)	1.016	0.392-2.633	0.975			
(-)	0.758	0.368-1.559	0.451			
Ki67			<0.001			0.005*
≤10	1.000	4.249-14.517		1.000		
>10	7.854	3.295-12.035	<0.001	2.633	1.276-5.433	0.009
Unknown	6.297	0.225-0.551	<0.001	3.034	1.480-6.223	0.002
RPR (>0.058vs≤0.058)	0.334	0.186-0.601	<0.001			
PVPR (>0.044vs≤0.044)	0.155	0.066-0.360	<0.001	0.281	0.117-0.675	0.005*
HPR (>0.607vs≤0.607)	0.155	0.066-0.360	<0.001			
LWR (>0.218vs≤0.218)	0.316	0.183-0.548	<0.001			
SIRI (>0.745vs≤0.745)	2.601	1.445-4.682	0.001			

*Statistically significant difference.

**Figure 4:**
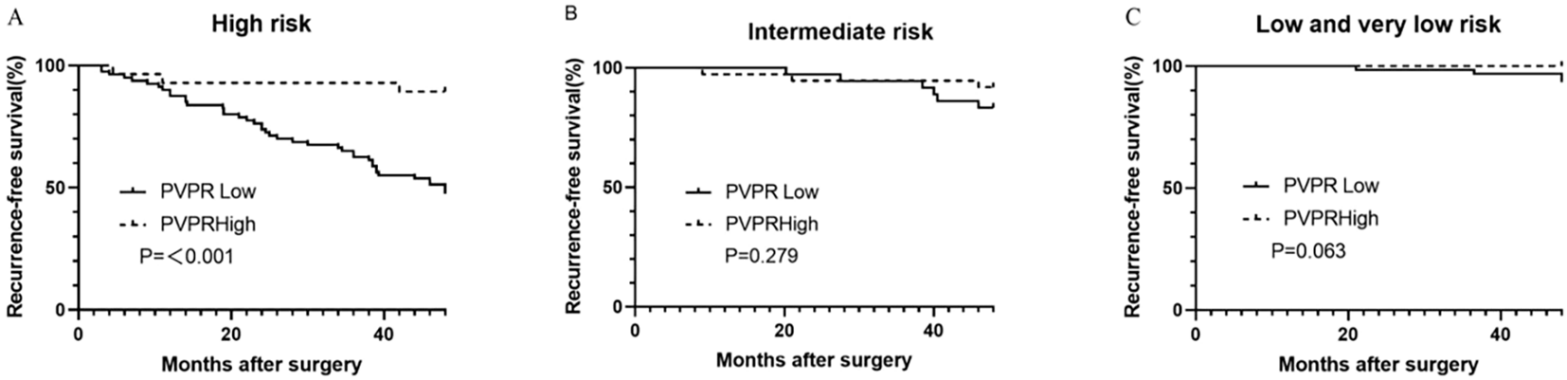
Kaplan–Meier survival curves for recurrence-free survival according to the PVPR in high- **(A)**, intermediate- **(B)**, and low- and very-low-risk **(C)** subgroups.

Our results from the subgroup analysis showed that patients in the high-PVPR group in the high-risk subgroup had prolonged RFS (P < 0.001); however, this was not the case in the intermediate subgroup and the low and very low subgroups (P = 0.279 and 0.063), respectively (**Figure 4**).

### 3.4 Correlation between inflammatory markers and clinical characteristics

Because of the strong correlations shown between PVPR and prognosis, the clinical characteristics of patients categorized by PVPR were adopted and displayed in **Table 3**. According to our research, there was a strong and positive association between PVPR and tumor size (P = 0.006), gastrointestinal bleeding (P < 0.001), mitotic index (P = 0.002), NIH risk category (P = 0.001), CD34 (P = 0.027), Ki-67 (P < 0.001), HPR (P < 0.001), and RPR (P < 0.001). Due to the lack of clear association between RPR, LWR, SIRI, HPR, and prognosis, there was no presentation of the patient features broken down by RPR, LWR, SIRI, and HPR.

**Table 3:** Relationship between PVPR and clinicopathological features in patients with GIST.

Characteristics	PVPR≤0.044 (n=178)	PVPR>0.044 (n=117)	χ^2^	P-value
Age (%)			0.220	0.639
≤60	97 (54.5)	67 (57.3)		
>60	81 (45.5)	50 (42.7)		
Sex (%)			1.134	0.287
Male	77 (43.3)	58 (49.6)		
Female	101 (56.7)	59 (50.4)		
Tumor site (%)			4.190	0.242
Stomach	117 (65.7)	81 (69.2)		
Colorectum	4 (2.2)	7 (6.0)		
Small intestine	43 (24.2)	23 (19.7)		
Other	14 (7.9)	6 (5.1)		
Tumor size (cm) (%)			12.435	0.006*
≤4.0	50 (28.1)	43 (36.8)		
4.1-6.0	41 (23.0)	32 (27.4)		
6.1-9.0	41 (23.0)	31 (26.5)		
>9.0	46 (25.8)	11 (9.4)		
Surgery (%)			0.156	0.693
Open	155 (87.1)	100 (85.5)		
Minimally invasive	23 (12.9)	17 (14.5)		
Gastrointestinal bleeding (%)			15.032	<0.001*
Yes	50 (28.1)	11 (9.4)		
No	128 (71.9)	106 (90.6)		
Mitotic index/50 HPF (%)			19.055	0.002*
≤5	122 (68.5)	103 (88.0)		
6-10	40(22.5)	14 (12.0)		
>10	10 (5.6)	0 (0.0)		
Unknown	6 (3.4)	0 (0.0)		
NIH risk category (%)			16.711	0.001*
High risk	80 (44.9)	28 (23.9)		
Intermediate risk	36 (20.2)	37 (31.6)		
Low risk	53 (29.8)	38 (32.5)		
Very low risk	9 (5.1)	14 (12.0)		
Histologic subtypes (%)			2.770	0.250
Spindle	135 (75.8)	79 (67.5)		
Epithelioid	4 (2.2)	5 (4.3)		
Mixed	39 (21.9)	33 (28.2)		
Adjuvant therapy (%)			0.055	0.973
Yes	73 (41.0)	47 (40.2)		
No	51 (28.7)	33 (28.2)		
Unknown	54 (30.3)	37 (31.6)		
CD117 (%)			4.211	0.092
(+)	174 (97.8)	109 (93.2)		
(+, partial)	3 (1.7)	7 (6.0)		
(-)	1 (0.5)	1 (0.8)		
CD34 (%)			7.229	0.027*
(+)	140 (78.7)	99 (84.6)		
(+, partial)	12 (6.7)	12 (10.3)		
(-)	26 (14.6)	6 (5.1)		
DOG1 (%)			0.695	0.734
(+)	166 (93.3)	112 (95.7)		
(+, partial)	5 (2.8)	2 (1.7)		
(-)	7 (3.9)	3 (2.6)		
SMA (%)			4.655	0.098
(+)	29 (16.3)	9 (7.7)		
(+, partial)	19 (10.7)	14 (12.0)		
(-)	130 (73.0)	94 (80.3)		
Ki-67 (%)			20.346	<0.001*
≤10	115 (64.6)	103 (88.0)		
>10	33 (18.5)	6 (5.1)		
Unknown	30 (16.9)	8 (6.8)		
HPR (%)			154.719	<0.001*
≤0.607	158 (88.8)	19 (16.2)		
>0.607	20 (11.2)	98 (83.8)		
RPR (%)			77.143	<0.001*
≤0.058	128 (71.9)	23 (19.7)		
>0.058	50 (28.1)	94 (80.3)		
LWR (%)			1.939	0.164
≤0.218	32 (18.0)	14 (12.0)		
>0.218	146 (82.0)	103 (88.0)		
SIRI (%)			2.684	0.101
≤0.745	74 (41.6)	60 (51.3)		
>0.745	104 (58.4)	57 (48.7)		

*Statistically significant difference.

### 3.5 Construction and validation of the nomogram

**Figure 5:**
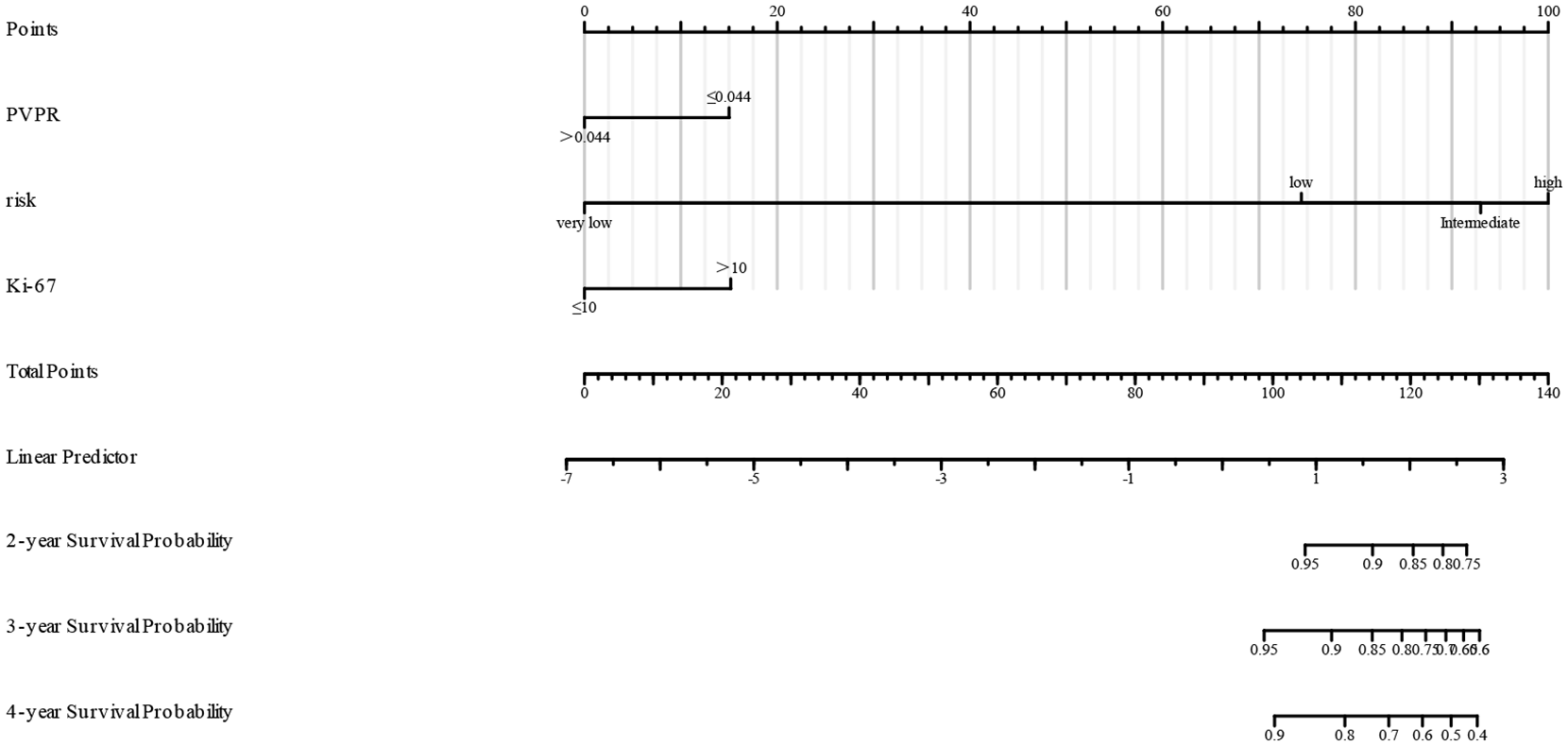
Nomogram to predict the probabilities of 2-, 3-, and 4-year recurrence-free survival (RFS) of primary GIST. The nomogram is based on NIH risk, mitotic index, and PVPR to predict the survival of GISTs, by summing the points of each independent factor, plotted on the “Total points” line, which corresponds to predictions of recurrence-free survival.

**Figure 6:**
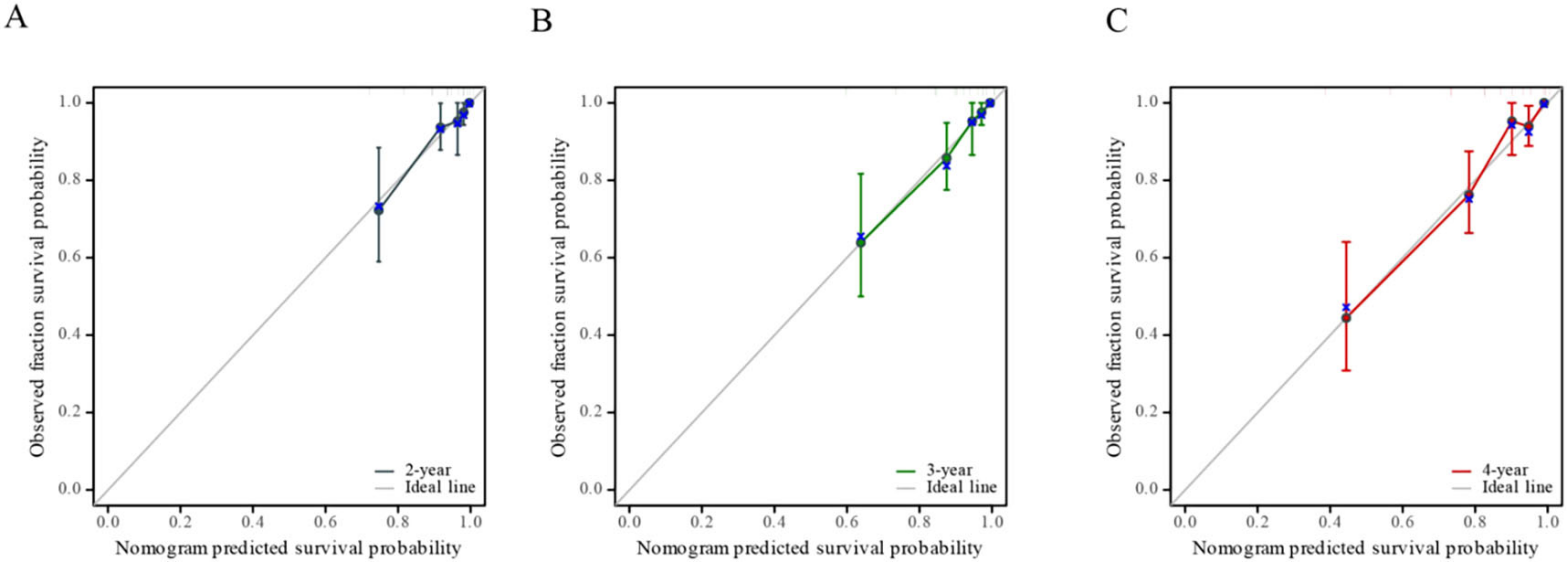
Calibration curve of nomogram-predicted recurrence-free survival (RFS), including the calibration curves of 2-year **(A)**, 3-year **(B)**, and 4-year **(C)** survival rates respectively.

**Figure 7:**
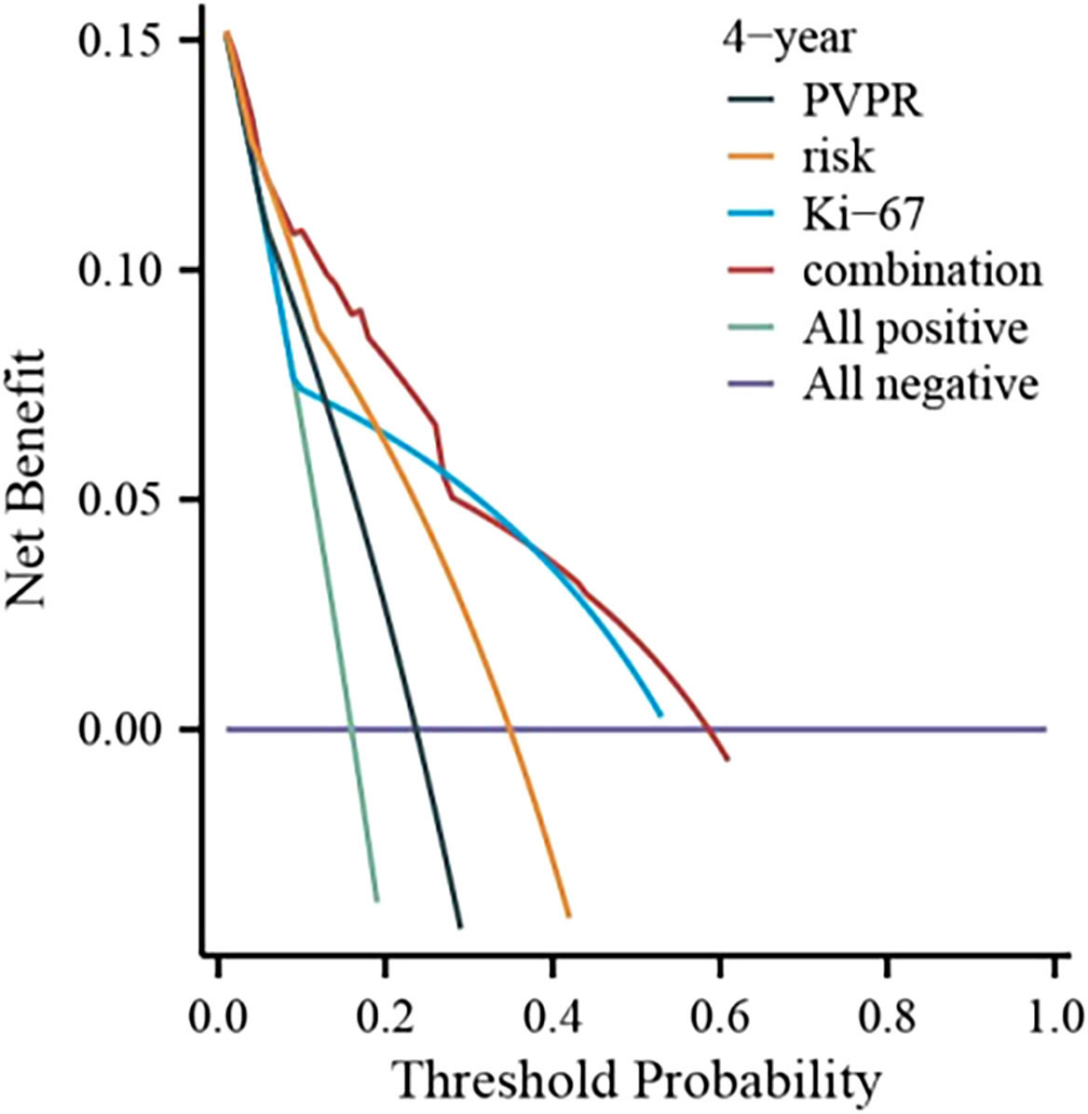
DCA curve of the nomogram.

We created a nomogram using the results of multivariate COX analysis, including PVPR, NIH risk categories, and Ki-67. We can forecast each patient’s 2-, 3-, and 4-year RFS probability by adding the scores for each variable (**Figure 5**). To evaluate its functionality, 1,000 bootstrap re-samples were performed on the nomogram for calibration plot–based internal validation. We also assessed how well the nomogram predicted the chance of recurrence following R0 resection of GISTs. The nomogram’s concordance probability was 0.823 (0.795–0.851). The calibration curve results of the prediction model show that the calibration curve of the 3-year survival rate is relatively close to the actual value, and the prediction model has high accuracy (p < 0.01) (**Figure 6**). Constructing decision curve analysis (DCA) for evaluating the clinical effectiveness of nomogram shows a good clinical effectiveness. The DCA constructed by the predicted model of 4 years after surgery is above the horizontal line at a threshold probability of 0–0.15, indicating the highest return over a large range (**Figure 7**). This model predicts patient prognosis and improves clinical outcomes.

## 4 Discussion

GIST is the most common in gastrointestinal sarcoma, and the most effective treatment method now is surgical resection. However, the recurrence and metastasis of primary diseases after surgery result in a shortened survival time. Therefore, predicting the risk of recurrence and metastasis can help early clinical screening of patients with a poor prognosis, timely intervention, and subsequent treatment, which is particularly important. Currently, numerous research works have found that systemic inflammation and local immune response influence tumor development and the survival of patients with cancer (9). The biological behavior of the occurrence, development, and invasion of malignant tumors depends on the malignant characteristics, in addition to the tumor microenvironment. Regarded as a crucial element of the tumor microenvironment, inflammatory cells facilitate invasion and metastasis by upsetting the immune system and ultimately allowing tumor cells to evade immune recognition (15, 18).

Up to now, multiple investigations have demonstrated a correlation between inflammatory biomarkers and prognosis of patients with GIST (16, 17, 19, 20), but, which index is better, there is no consistent conclusion. Li et al. found that platelet-to-lymphocyte ratio, SIRI is an independent risk factor of recurrence after surgery (18, 21). Monocyte-to-lymphocyte ratio and neutrophil-to-lymphocyte ratio are also confirmed as independent prognostic factors for disease-free survival in GISTs (14, 19). Some nutritional indicators such as Geriatric Nutrition Risk Index (20, 22) are considered as a prognostic factor (21, 23).

MPV and PC are the two most significant platelet indices, and, lately, its integration into the PVPR index has been applied to the assessment of patients’ prognoses for malignant tumors. According to Gu et al., Hepatocellular Carcinoma (HCC) patients with low MPV/PC had noticeably better overall survival (14, 24). Poor ratios of MPV/PC were linked to poor cancer-specific survival, according to Feng et al. (22, 25). In colorectal cancer, Wu et al. demonstrated the predictive utility of MPV/PC (23, 26). In cervical cancer, the patients with low MPV/PC had a considerably better overall survival than those who had high MPV/PC, and MPV/PC was a stand-alone predictive marker for cervical cancer (24, 27). However, the predictive role of PVPR in patients with GIST has not been explored yet. So, we conducted a retrospective study on patients with GIST after R0 resection and mainly compared the predictive ability of five inflammatory indicators—LWR, HPR, SIRI, PVPR, and RPR—in RFS of GISTs.

In our research, we tested their ROC curves; these indicators all have predictive significance (p < 0.05), but the AUC of PVPR is maximum, which reached 0.721, representing its good predictive function for RFS. Kaplan–Meier curves also indicated that RFS was significantly better in the high-PVPR group. Whether in univariate or multivariate analysis, compared with other inflammatory biomarkers such as LWR, HPR, SIRI, and RPR, PVPR was an independent prognostic indicator for patients with GIST. Although LWR, HPR, SIRI, and RPR are associated with RFS univariate analysis, they are not independent prognostic indicators. However, in subgroup analysis, only in the patients with high risk, PVPR has shown a good ability to distinguish patients with different prognosis. These results demonstrated that PVPR was a better inflammatory indicator than other ones for predicting survival in patients with GIST. In the comparison of PVPR with baseline clinical features, we also found that PVPR was significantly related to tumor size, gastrointestinal bleeding, mitotic index, NIH risk category, CD34, and Ki-67.

We have now confirmed that PVPR can predict the prognosis of GISTs, but the mechanism has not been fully elucidated. It might be connected to the following elements. Firstly, PVPR is calculated by MPV and PC. Platelets are discoid-shaped fragments derived from bone marrow megakaryocytes (25, 28). Although not strictly appointed within the inflammatory pathway, the platelet can be viewed as an extension of the cellular immune system, and the platelet in the middle of diverse inflammatory processes influences normal leukocyte biology and inflammatory signals (26, 29). Platelets get activated and aggregated in response to circulating tumor cells (CTCs), and these activated platelets collect and shield CTCs from natural killer cells and shear stress. Last but not least, platelets promote CTC resistance to anoikis, angiogenesis, extravasation, and, ultimately, metastasis (27). MPV has been shown to be a good predictor of platelet activity, and it is linked to a number of prothrombotic and proinflammatory illnesses (28, 31). In physiological conditions, MPV is inversely proportional to the PC, which is associated with hemostasis maintenance and preservation of constant platelet mass (29, 32). Therefore, the ratio of MPV to PC is often studied as a whole. Secondly, research had shown that patients with cancer have a higher PC and are more likely to experience thromboembolism, which is linked to significant morbidity and death. Indirectly acting on endothelial cells or leukocytes, cancer cells can also stimulate platelets by the contact of a membrane protein on their surface with a particular receptor on platelets to promote thrombus formation. They release proinflammatory cytokines that stimulate prothrombotic alteration in endothelial cells, such as interleukin-1b and tumor necrosis factor–a (30, 33). In other words, platelets are not only a consequence of malignant tumors but also a part of their development process. In general, platelet wear during coagulation may cause the count to drop, but proinflammatory cytokines can activate megakaryocytes, which can result in a significant increase in thrombocyte synthesis and release. Platelet parameters can serve as diagnostic indicators for certain disorders because they exhibit distinct variations (28, 31).

At the end of this study, we incorporated the independent prognostic factors such as PVPR, NIH risk category, and Ki-67 to assemble the nomogram. The nomogram demonstrated a high degree of accuracy in predicting GIST survival (C-index = 0.823). More investigation is required to enhance the nomogram through the analysis of more thorough prognostic data, and this model’s efficacy need to be assessed in upcoming clinical uses.

The significance of our research lies in finding an auxiliary prediction tool that can be used in addition to mitotic index, Ki-67, and danger levels. It is simple and easy to obtain and has the potential to be applied in clinical work in the future. Antiplatelet drugs may lower infection-related mortality, according to recent research, which raises the possibility that altering platelet responses to inflammation has therapeutic value (25, 28). So, we look forward to more research and application of antiplatelet drugs in gastrointestinal tumors in the future.

There are several restrictions on this study. To begin with, our study had a medium sample size and was a retrospective single-center investigation. Due to limited data, it is not possible to investigate 5-year RFS, and there is a lack of longitudinal comparative data, which may result in a certain degree of deviation. Secondly, the study included patients who accept TKI adjuvant therapy after surgery, and it could significantly prolong RFS. Thirdly, the nomogram was lack of external validation, which may lead to inaccurate models.

## 5 Conclusion

Through the above research, we found that PVPR is an independent prognostic indicator for RFS after GIST R0 resection, especially for high-risk patients. It has superior prognostic ability than LWR, HPR, SIRI, and RPR. Meanwhile, PVPR is easy to obtain, is cost-effective, and has good predictive performance. PVPR > 0.044 can help predict recurrence and assess patient risk stratification.

## Data Availability

The raw data supporting the conclusions of this article will be made available by the authors, without undue reservation.
